# Association of Rest-Activity Rhythm and Risk of Developing Dementia or Mild Cognitive Impairment in the Middle-Aged and Older Population: Prospective Cohort Study

**DOI:** 10.2196/55211

**Published:** 2024-05-07

**Authors:** Shahab Haghayegh, Chenlu Gao, Elizabeth Sugg, Xi Zheng, Hui-Wen Yang, Richa Saxena, Martin K Rutter, Michael Weedon, Agustin Ibanez, David A Bennett, Peng Li, Lei Gao, Kun Hu

**Affiliations:** 1 Massachusetts General Hospital Boston, MA United States; 2 Harvard Medical School Boston, MA United States; 3 Broad Institute Cambridge, MA United States; 4 Brigham and Women's Hospital Boston, MA United States; 5 Faculty of Medicine, Biology and Health University of Manchester Manchester United Kingdom; 6 Manchester University NHS Foundation Trust Manchester Academic Health Science Centre NIHR Manchester Biomedical Research Centre Manchester United Kingdom; 7 University of Exeter Exeter United Kingdom; 8 Adolfo Ibañez University Santiago Chile; 9 RUSH University Chicago, IL United States

**Keywords:** circadian rhythm, dementia, actigraphy, cognitive decline, RAR, rest-activity rhythms, cognitive impairment

## Abstract

**Background:**

The relationship between 24-hour rest-activity rhythms (RARs) and risk for dementia or mild cognitive impairment (MCI) remains an area of growing interest. Previous studies were often limited by small sample sizes, short follow-ups, and older participants. More studies are required to fully explore the link between disrupted RARs and dementia or MCI in middle-aged and older adults.

**Objective:**

We leveraged the UK Biobank data to examine how RAR disturbances correlate with the risk of developing dementia and MCI in middle-aged and older adults.

**Methods:**

We analyzed the data of 91,517 UK Biobank participants aged between 43 and 79 years. Wrist actigraphy recordings were used to derive nonparametric RAR metrics, including the activity level of the most active 10-hour period (M10) and its midpoint, the activity level of the least active 5-hour period (L5) and its midpoint, relative amplitude (RA) of the 24-hour cycle [RA=(M10-L5)/(M10+L5)], interdaily stability, and intradaily variability, as well as the amplitude and acrophase of 24-hour rhythms (cosinor analysis). We used Cox proportional hazards models to examine the associations between baseline RAR and subsequent incidence of dementia or MCI, adjusting for demographic characteristics, comorbidities, lifestyle factors, shiftwork status, and genetic risk for Alzheimer's disease.

**Results:**

During the follow-up of up to 7.5 years, 555 participants developed MCI or dementia. The dementia or MCI risk increased for those with lower M10 activity (hazard ratio [HR] 1.28, 95% CI 1.14-1.44, per 1-SD decrease), higher L5 activity (HR 1.15, 95% CI 1.10-1.21, per 1-SD increase), lower RA (HR 1.23, 95% CI 1.16-1.29, per 1-SD decrease), lower amplitude (HR 1.32, 95% CI 1.17-1.49, per 1-SD decrease), and higher intradaily variability (HR 1.14, 95% CI 1.05-1.24, per 1-SD increase) as well as advanced L5 midpoint (HR 0.92, 95% CI 0.85-0.99, per 1-SD advance). These associations were similar in people aged <70 and >70 years, and in non–shift workers, and they were independent of genetic and cardiovascular risk factors. No significant associations were observed for M10 midpoint, interdaily stability, or acrophase.

**Conclusions:**

Based on findings from a large sample of middle-to-older adults with objective RAR assessment and almost 8-years of follow-up, we suggest that suppressed and fragmented daily activity rhythms precede the onset of dementia or MCI and may serve as risk biomarkers for preclinical dementia in middle-aged and older adults.

## Introduction

Dementia represents a major public health concern with profound social, economic, and health care implications, and it is a leading cause of disability and dependency among the older adult population. It is estimated that over 55 million individuals worldwide are affected by dementia [1]. As the global population ages, understanding the etiology and potential risk factors associated with dementia has become a critical area of research. While the pathogenesis of dementia remains multifactorial and complex, recent studies have underscored the link of disrupted daily rest-activity rhythms (RARs) to cognitive decline and the development of dementia in older adults [2,3].

The RAR is governed by the circadian system and interacts with the daily cycles of behavioral and environmental changes, which is crucial for maintaining optimal physiological functioning and coordination of bodily processes [4]. The circadian system consists of a network of circadian clocks in the brain and peripheral organs, and these clocks can be affected or reset by environmental conditions, work schedules and social patterns via different time cue inputs including light exposure, food intake, and physical activity [5]. The importance of well-functioning circadian regulation in overall health has been widely recognized. Studies have demonstrated that disruptions in the RAR are associated with various adverse health conditions, including cancer [6], cardiovascular diseases [7], digestive diseases [6], respiratory diseases [6,8], and depression [9]. In recent years, emerging evidence has suggested that disruptions in the RAR could contribute to neurodegenerative processes and cognitive impairment [2,10,11]. However, there are notable limitations in previous studies such as small sample size [12-17], cross-sectional design [10,18-27], focusing on only older population [12,14-17], and short follow-up duration [14,15].

In this study, we used data from more than 94,000 participants aged 43-79 years in a large prospective cohort study, the UK Biobank (UKB), with at least 6 continuous days of wrist actigraphy recording and up to 7.5 years of follow-up to examine the association of RAR measures with future risk of dementia or mild cognitive impairment (MCI). We also explored the potential effects of age, genetics, and shiftwork on this association. We hypothesized that disrupted RAR patterns such as suppressed and fragmented 24-hour activity rhythms are associated with a higher risk of developing dementia or MCI.

## Methods

### Study Population and Data Source

We used longitudinal data on UKB participants (age range at baseline 43-79 years; 54% female) [28]. Upon enrollment, UKB participants completed a series of questionnaires that collected their demographic, lifestyle, and medical history information. Participants consented to releasing their electronic health records from the United Kingdom’s centralized National Health Service (NHS), which were then stored in the UKB’s Hospital Inpatient Data library [29]. In the UKB cohort, 103,711 participants completed actigraphy assessments between 2013 and 2015 (2.8 to 9.7 years after enrollment) [29]—the baseline of this study. We used follow-up data until September 2021 (maximum and median follow-up after actigraphy: 7.5 and 5 years, respectively). After excluding those participants with poor calibration of activity counts, significant gaps in data likely due to off-wrist periods, <6 days of collected data, dementia or MCI at baseline, or any missing covariate, 91,517 participants were included in this study.

### Ethical Considerations

The UKB received approval from the North West Multi-centre Research Ethics Committee (11/NW/03820; 16/NW/0274; 21/NW/0157). This study was conducted under the terms of the UKB (33883) and Mass General Brigham Institutional Review Board (#2018P000356).

### Assessment of RARs

Participants wore triaxial accelerometer devices (Axivity AX3; Axivity Ltd) for up to 7 days during the collection period. Prior actigraphy assessment of older adults [30,31] and existing criteria from the UKB [32] were used to perform quality checks. Activity counts in each 15-second epoch were derived from accelerometer data sampled at ~100 Hz (see Multimedia Appendix 1 [29,33]). The first 6 days of activity counts were used to obtain the following nonparametric RAR measures [34]: (1) activity counts during the most active 10-hour period of the 24-hour cycle (M10) and (2) the midpoint of the M10 period (M10 midpoint); (3) activity counts during the least active 5-hour period of the 24-hour cycle (L5)—likely representing hours during sleep, and (4) the timing midpoint of the L5 period (L5 midpoint); (5) relative amplitude (RA) calculated as (M10-L5)/(M10+L5)—representing the robustness of a 24-hour rest-activity cycle; (6) interdaily stability (IS) that quantifies the stability of the 24-hour rhythm between different days (Multimedia Appendix 2); and (7) intradaily variability (IV) that describes the fragmentation of the rhythm (Multimedia Appendix 2). Cosinor analysis was also performed to derive 2 additional measures of 24-hour activity rhythms: the amplitude (midline to peak) and acrophase (time of the peak) of the 24-hour rhythm. All RAR data analyses were performed using the eZActi2 software [35,36].

### Assessment of Dementia and MCI

Study participant hospitalization records were kept within the UK’s NHS during the follow-up period before being released by the UKB. The UKB provided algorithmically defined incidence of health matters from *ICD-10* (*International Classification of Disease, 10th Revision*) codes. We obtained data from clinical coding of dementia (*ICD-10* code: F05) and MCI (*ICD-10* code: F0.67), and from the UKB algorithm “date of all-cause dementia” (field 42018). Age at death or the date of death was based on the death certificates in the NHS. The first occurrence of dementia or MCI (time-to-event) was the first date of diagnosis relative to the actigraphy assessment date.

### Assessment of Covariates

The following covariates that may affect RARs were considered in this study: (1) demographics, including age at actigraphy, male or female designated sex of individuals, self-reported ethnicity as European or non-European, college-level education (reported as yes or no), and the Townsend deprivation index; (2) comorbidities, including sleep apnea (based on *ICD-10* code G47.30), circulatory disease (based on reports of high cholesterol, diabetes, hypertension, ischemic heart disease, smoking, and peripheral vascular disease), BMI >30, and morbidity burden (classified at the time of actigraphy as none, moderate, or high based on previously used methods that summed the presence of diseases or disorders of the endocrine, connective tissue, gastrointestinal, hematological, musculoskeletal, immune, renal, and respiratory systems as well as any cancers) [37-40]; (3) lifestyle, including alcohol intake (categorized by daily use, 3-4 times per week, 1-2 times per week, a few times per month, and never), smoking status (categorized as current, previous, and never); (4) shiftwork (yes or no); and (5) genetics based on the polygenic risk score (PRS) for Alzheimer disease. We calculated the single PRS for Alzheimer disease using the PRS continuous shrinkage [41] method. This method calculates posterior effect size from genome-wide association study summary statistics with models comprising information of local linkage disequilibrium patterns, and thus reduces PRS error and improves performance. In this study we used genome-wide association study summary statistics from a recent study for Alzheimer disease [42], and the linkage disequilibrium reference panel matrices from the UKB. The PRS continuous shrinkage default settings were used, and after deriving the posterior summary statistics, we used PLINK2 [43,44].

### Statistical Analysis

Cox proportional hazard models were used to assess the associations of RAR measures with the subsequent incidence of dementia or MCI. The results were reported as hazard ratios (HRs) with corresponding 95% CIs. For each RAR measure (except M10 midpoint, L5 midpoint, and phase), participants were divided into 4 quartiles (Q1-Q4). The highest quartile (Q4) was used as a reference for RA, M10, IS, and 24-hour amplitude; the lowest quartile (Q1) was used as a reference for L5 and IV. These reference levels were chosen based on prior findings and our hypothesis regarding the direction of the association of each RAR measure with the risk of dementia and MCI, that is, the level of the RAR measure that is hypothesized to be linked to the lowest risk of developing dementia was considered as the reference [37]. Acrophase, M10 midpoint, and L5 midpoint were categorized into 3 tertiles: earlier (6:00 AM-1:34 PM for acrophase, 6:00 AM-12:58 PM for M10 midpoint, and noon-2:47 AM for L5 midpoint), middle (1:34 PM-2:25 PM for acrophase, 12:58 PM-2:01 PM for M10 midpoint, and 2:47 AM-3:44 AM for L5 midpoint), and later (2:25 AM-6:00 AM for acrophase, 2:01 AM-6:00 AM for M10 midpoint, and 3:44 AM-noon for L5 midpoint) groups, and the middle groups were used as the reference [14,15,17]. Separate Cox models were also used to obtain HRs for 1 SD change in each RAR measure. Secondary analyses were performed to investigate (1) the associations between RAR measures and the risk of developing dementia (by excluding participants who only developed MCI); (2) the associations between RAR measures and risk of developing dementia or MCI after excluding those participants who were shift workers at baseline; (3) the interaction effects of Alzheimer disease PRS (<median PRS vs >median PRS) and RAR measures on the risk of developing dementia or MCI (by including interaction terms and also stratifying participants based on their PRS values); and (4) the interaction effects of age (<70 vs ≥70 years) and RAR measures on the risk of developing dementia or MCI (by including interaction terms and also stratifying participants based on their age). All statistical analyses were performed using JMP Pro (version 16, SAS Institute).

## Results

### Participant Characteristics

Table 1 describes the demographic, lifestyle, and clinical comorbidity data gathered from the 91,517 UKB participants who were included in this study. Most participants were of White European descent (>95%). In comparison to the 90,962 participants who did not develop dementia or MCI, those who did (n=555) were older (69.6 vs 62.4 years) and more likely to be male (n=308, 55.5% vs n=39,758, 43.7%), had lower levels of education (n=215, 38.7% vs n=39,372, 43.3% attended college), had a higher prevalence of sleep apnea (n=10, 1.8% vs n=771, 0.8%), and circulatory system disease (n=261, 47% vs n=22,273, 24.4%), had a higher morbidity burden (2.0, SD 1.8 vs 1.1, SD 1.3), and were more likely to be current or past smokers (n=40, 7.2% vs n=6271, 6.9% and n=342, 61.6% vs n=48,428, 53.2%).

**Table 1:** Baseline demographics, lifestyle, and clinical comorbidities of participants (n=91,517), by dementia or MCI^a^ status at follow-up^b^.

					Participants who developed dementia or MCI (n=555)			Participants who did not develop dementia or MCI (n=90,962)
**Demographics^c^**								
	Age at actigraphy (years), mean (SD)			69.6 (5.4)			62.4 (7.8)	
	**Sex, n (%)**							
		Male	308 (55.5)			39,758 (43.7)		
		Female	247 (44.5)			51,204 (56.3)		
	Attended college, n (%)			215 (38.7)			39,372 (43.3)	
	Townsend deprivation index (higher)^d^, n (%)			274 (49.4)			45,007 (49.5)	
	European ethnic background			535 (96.4)			87,951 (96.7)	
**Rest-activity rhythmicity characteristics,** **mean (SD)**								
	Relative amplitude			0.953 (0.047)			0.956 (0.035)	
	Amplitude (24-hour, AU^e^)			28.0 (15.9)			33.3 (15.9)	
	M10^f^ (count^g^)			127,003 (58,873)			149,468 (61,082)	
	L5^h^ (count)			2708 (2622)			2367 (2565)	
	Phase (hours after midnight)			13.79 (1.18)			14.02 (1.24)	
	IV^h^, AU			0.95 (0.24)			0.91 (0.24)	
	IS^i^, AU			0.54 (0.13)			0.52 (0.13)	
**Comorbidities**								
	Sleep apnea, n (%)			10 (1.8)			771 (0.8)	
	Circulatory system disease, n (%)			261 (47)			22,273 (24.4)	
	BMI > 30 kg/m^2^, n (%)			125 (22.5)			17,586 (19.3)	
	Morbidity burden (number of diagnoses), mean (SD)			2.0 (1.8)			1.1 (1.3)	
	**Alcohol intake, n (%)**							
		Daily	160 (28.8)			20,833 (22.9)		
		3 to 4 times per week	117 (21.1)			23,643 (26.0)		
		Once or twice per week	104 (18.7)			22,830 (25.1)		
		Few times per month	114 (20.5)			18,522 (20.4)		
		Never	60 (10.8)			5134 (5.6)		
	**Smoking status, n (%)**							
		Current	40 (7.2)			6271 (6.9)		
		Previous	342 (61.6)			48,428 (53.2)		
		Never	173 (31.2)			36,263 (39.9)		
	Shiftwork, n (%)			18 (3.2)			7351 (8.1)	

^a^MCI: mild cognitive impairment.

^b^Cardiovascular disease means the presence of any of the following: hypertension, high cholesterol, smoking, diabetes, ischemic heart disease, and peripheral vascular disease.

^c^Data come from recruitment between 2.8 and 9.7 years before actigraphy.

^d^Participants that scored above the median Townsend deprivation index.

^e^AU: arbitrary unit.

^f^M10: activity level of the most active 10-hour period.

^g^Count: relative mean change in acceleration.

^h^L5: activity level of the least active 5-hour period.

^i^IV: intradaily variability.

^j^IS: interdaily stability.

### RARs and Incident Dementia or MCI

**Figure 1:**
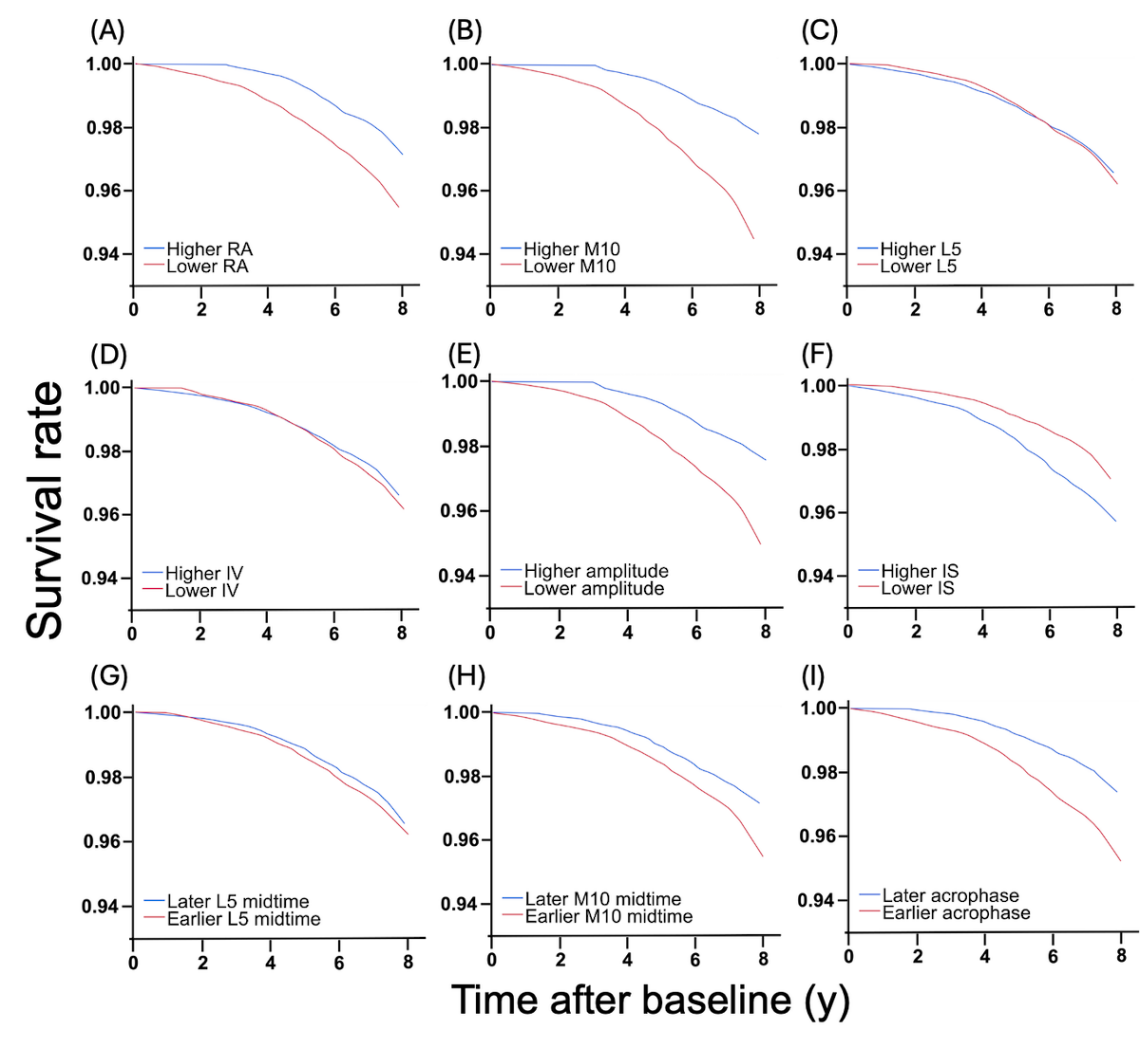
Rest-activity rhythm and risk of developing dementia or MCI. Survival curves for dementia or MCI since baseline (actigraphy assessment) for (A) participants with lower RA (1st quartile) and higher RA (4th quartile), (B) participants with lower M10 (1st quartile) and higher M10 (4th quartile), (C) participants with lower L5 (1st quartile) and higher L5 (4th quartile), (D) participants with lower IV (1st quartile) and higher IV (4th quartile), (E) participants with lower amplitude (1st quartile) and higher amplitude (4th quartile), (F) participants with lower IS (1st quartile) and higher IS (4th quartile), (G) participants with earlier L5 midpoint (1st tertile) and later L5 midpoint (3rd tertile), (H) participants with earlier M10 midpoint (1st tertile) and later M10 midpoint (3rd tertile), and (I) participants with earlier acrophase (1st tertile) and later acrophase (3rd tertile). IS: interdaily stability; IV: intradaily variability; L5: activity level of the least active 5-hour period; M10: activity level of the most active 10-hour period; MCI: mild cognitive impairment; RA: relative amplitude.

Table 2 presents multivariable-adjusted HRs for dementia or MCI associated with RAR metrics when considered as quartiles of exposure or per SD difference. Figure 1 shows survival plots for incident dementia or MCI associated with RAR metrics. The risk of dementia or MCI was statistically higher in those with more suppressed and fragmented 24-hour activity rhythms as quantified by lower RA (multivariable-adjusted HR per 1-SD decrease=1.23, 95% CI 1.16-1.29; Q1 vs Q4, HR 1.88, 95% CI 1.46-2.41; Figure 1A), lower M10 (multivariable-adjusted HR per 1-SD decrease=1.28, 95% CI 1.14-1.44; Q1 vs Q4, HR 1.69, 95% CI 1.30-2.19; Figure 1B), higher L5 (multivariable-adjusted HR per 1-SD increase=1.15, 95% CI 1.10-1.21; Q4 vs Q1, HR 1.51, 95% CI 1.19-1.91; Figure 1C), and larger IV (multivariable-adjusted HR per 1-SD increase=1.14, 95% CI 1.05-1.24; Q4 vs Q1, HR 1.56, 95% CI 1.20-2.02; Figure 1D). Consistently, the risk of dementia or MCI was statistically higher in those with smaller 24-hour amplitude based on cosinor analysis (multivariable-adjusted HR per 1-SD decrease=1.32, 95% CI 1.17-1.49; Q1 vs Q4, HR 1.86, 95% CI 1.42-2.42; Figure 1E). In addition, participants with delayed L5 midpoint had a lower risk for dementia or MCI (multivariable-adjusted HR per 1-SD increase or ~75 min delay in L5 midpoint=0.92, 95% CI 0.85-0.99; Figure 1G). IS (Figure 1F), M10 midpoint (Figure 1H), and acrophase (Figure 1I) had no significant associations with the risk of dementia or MCI.

In addition, we found that the risk was higher for older participants (multivariable-adjusted HR for each year older at baseline=1.17, 95% CI 1.15-1.19) and male participants (multivariable-adjusted HR 1.33, 95% CI 1.11-1.58).

The associations between RAR measures and incident dementia or MCI remained similar when including only participants who developed dementia (Multimedia Appendix 3) or when excluding those shift workers (Multimedia Appendix 4). In addition, the associations between RAR metrics and risk of dementia or MCI were independent of PRS (*P* values >.10 for the interaction terms of PRS and all RAR measures; Multimedia Appendix 5) while higher PRS was associated with an increased risk of dementia or MCI (multivariable-adjusted HR per 1-SD increase=1.48, 95% CI 1.36-1.60). Moreover, the RAR-dementia or MCI associations appeared to be similar for the younger (<70 years old) and older (≥70 years old) participants (*P* values >.10 for the interaction terms of age group and all RAR measures, except M10; Multimedia Appendix 6).

**Table 2:** Relationships of RAR^a^ measures with risk of developing dementia or MCI^b,c^.

RAR characteristics		Adjusted hazard ratio (95% CI)
**Relative amplitude**		
	Q1^d^	1.88 (1.46-2.41)
	Q2	1.29 (0.99-1.67)
	Q3	0.94 (0.71-1.26)
	Q4	Reference
	Per 1-SD decrease	1.23 (1.16-1.29)
**M10^e^**		
	Q1	1.69 (1.30-2.19)
	Q2	1.11 (0.83-1.47)
	Q3	1.03 (0.76-1.38)
	Q4	Reference
	Per 1-SD decrease	1.28 (1.14-1.44)
**M10 midpoint**		
	Earlier	1.10 (0.90-1.34)
	Middle	Ref
	Later	1.03 (0.83-1.27)
	Per 1-SD increase	0.93 (0.84-1.03)
**L5^f^**		
	Q1	Reference
	Q2	1.23 (0.96-1.57)
	Q3	1.00 (0.78-1.30)
	Q4	1.51 (1.19-1.91)
	Per 1-SD increase	1.15 (1.10-1.21)
**L5 midpoint**		
	Earlier	1.06 (0.87-1.30)
	Middle	Reference
	Later	0.86 (0.70-1.06)
	Per 1-SD increase	0.92 (0.85-0.99)
**IV^g^**		
	Q1	Reference
	Q2	1.64 (1.27-2.12)
	Q3	1.37 (1.05-1.78)
	Q4	1.56 (1.20-2.02)
	Per 1-SD increase	1.14 (1.05-1.24)
**IS^h^**		
	Q1	1.00 (0.78-1.27)
	Q2	0.82 (0.64-1.05)
	Q3	1.01 (0.81-1.26)
	Q4	Reference
	Per 1-SD decrease	0.98 (0.89-1.07)
**Amplitude**		
	Q1	1.86 (1.42-2.42)
	Q2	1.28 (0.96-1.70)
	Q3	1.22 (0.91-1.63)
	Q4	Reference
	Per 1-SD decrease	1.32 (1.17-1.49)
**Acrophase**		
	Earlier	0.99 (0.81-1.20)
	Middle	Reference
	Later	0.90 (0.73-1.13)
	Per 1-SD increase	0.93 (0.85-1.02)

^a^RAR: rest-activity rhythm.

^b^MCI: mild cognitive impairment.

^c^Models are adjusted for age at the time of actigraphy, sex, education, Townsend deprivation index, ethnic background, obesity, sleep apnea, morbidity burdens, circulatory disorders, night shiftwork status, alcohol intake, smoking status, and polygenic risk score of Alzheimer disease.

^d^Q: quartile.

^e^M10: activity level of the most active 10-hour period.

^f^L5: activity level of the least active 5-hour period.

^g^IV: intradaily variability.

^h^IS: interdaily stability.

## Discussion

### Principal Findings

In this large, prospective cohort study, we evaluated the association between RAR metrics, derived from wrist actigraphy, and incidence of dementia or MCI during a follow-up of up to 7.5 years. Our results underscore the significance of specific RAR metrics, notably RA, M10, amplitude, L5, L5 midpoint, and IV in delineating the dementia or MCI risk, independent of previously identified risk factors for dementia or cognitive decline.

Unlike previous studies, our analysis is based on a notably large sample size of >94,000 participants, using objective actigraphy assessments for RAR and spanning a broad age range of 43-79 years. This study adopted a longitudinal design with nearly 8 years of follow-up and comprehensively adjusted for known confounders, including demographic, shiftwork status, lifestyle, comorbidity, and genetics, to enhance the robustness of the results. Specifically, we showed that suppressed 24-hour rhythmicity (lower RA and 24-hour amplitude), accompanied by reduced activity levels (M10) during the active phase and increased activity levels during the resting phase (L5) as well as fragmented 24-hour rhythms (greater IV), were linked to the higher risk for dementia or MCI. These results are consistent with previous studies. For instance, using the Rush Memory and Aging Project data, the risk for Alzheimer dementia was higher in those with lower 24-hour amplitude and greater IV [12]; a longitudinal study of 2496 older men indicated that a larger increase in IV over a span of 7.5 years was associated with a steeper decline in Modified Mini-Mental State Examination scores [16]; and another study of 763 older women showed that reduction in RA was associated with elevated risks for MCI and probable dementia [17]. It is worth noting that our study showed consistent adverse impacts of disrupted rest-active rhythms across different age groups (≥70 years and <70 years).

Regarding the mechanisms underlying the RAR-dementia link, disturbances in circadian regulation and sleep-wake cycles have been proposed as one of the common pathological pathways [3]. Supporting this concept, shift work, an established major cause of circadian or sleep disturbances, has been linked to a higher risk for developing dementia [45-47]; and Musiek et al [22] identified a relationship between increased IV and amyloid plaque pathology in preclinical Alzheimer disease. Clearly, circadian disturbances not only occur in shift workers but also may be caused by other factors such as traveling in different time zones and social jet lag [48,49]. Consistently, we found that the associations of RAR disturbances with dementia risk remained in non–shift workers.

One “unexpected” result was the nonsignificant association of the stability measure (IS) of 24-hour rhythms with the risk for dementia or MCI because the reduction in IS has been linked to aging and dementia [12,26]. Notably, while other studies have similarly reported a lack of significant associations [13,14,17], our research contributes to more definitive insights into these complex associations with a substantially larger sample size. In a related study, Park et al [50] reported higher IS in older adults when compared to younger adults, and interpreted the results as the consequence of changes in daily schedules. This study raises a potential concern about the masking effect of daily schedules on RAR measures, especially IS [51]. An important follow-up question is how reliable IS can be in reflecting intrinsic changes in circadian regulation or predicting or capturing the long-term impacts of acute disturbed 24-hour behavioral cycles on circadian health and related cognitive changes. Future studies including circadian rhythms of other physiological variables or mathematical modeling for estimation of circadian rhythms [52-56] are needed to address the question.

The relationship between the RAR phase and dementia or MCI risk is still inconclusive. Our study identified earlier L5 mid-time as a risk factor for dementia or MCI, but not changes in acrophase or M10 mid-time. L5 mid-time is usually related to the timing of sleep, whereas M10 mid-time and acrophase are usually related to the timing of peak activity. Previous research has yielded varied results. For example, Lysen et al [13] did not observe any association between the circadian phase measured by L5 onset and risk of dementia or MCI, whereas Posner et al [14] observed an association between earlier L5 midpoint (but not M10 midpoint) with higher risk for dementia (but not MCI) [14]. Xiao et al [17] reported a significant linear association of delayed acrophase, M10, and L5 midpoints with a higher risk for dementia or MCI in older women. These inconsistent findings might be explained by unadjusted confounders that influence sleep timing, such as chronotype (ie, preferred sleep time), sleep disorders (eg, insomnia), use of sleep medication, and photoperiod. Future studies should consider controlling for such factors when clarifying relationships between the RAR phase and dementia or MCI.

### Clinical Implications and Future Research

Our findings provide insights into the clinical practice and future research in dementia and MCI prevention, screening, and intervention. Specifically, incorporating assessments of sleep and rest-activity patterns into routine health evaluations might be beneficial for middle-aged and older adults. In geriatric care, routine monitoring and management of RAR may help evaluate the factors affecting cognitive health. Educating caregivers and family members about the importance of consistent rest-activity patterns could be incorporated into the home-based care for individuals at risk. Tailoring preventive and therapeutic strategies to individuals based on their RAR characteristics, especially in populations like shift workers, could also be effective. Future research should further clarify the causality of the associations between RAR and cognition, and test whether interventions that improve sleep hygiene, modify light exposure, or adjust physical activity levels can positively impact RAR and, consequently, help prevent or slow cognitive decline.

### Strengths and Limitations

The strengths of this study include having a large sample size of more than 94,000 participants; using objective assessments of RAR using actigraphy; controlling for a large number of confounders, including demographic, lifestyle, comorbidity, genetics, and morbidity burden; large age range of participants (aged between 43 and 79 years); and the longitudinal study design with nearly 8 years of follow-up. Limitations of our study are as follows: (1) the majority of participants were of White European descent (>95%), limiting our ability to investigate racial or ethnic differences in the associations; (2) the rate of dementia or MCI events (~550 out of 94,000) appeared to be relatively low due to the overall young age of the participants (median age 63.5 years); (3) we were unable to differentiate between different types of dementia. However, this provides a great opportunity for future studies, when the participants became older, to investigate the long-term association of RAR and different types of dementia or MCI; (4) single-time assessment of actigraphy and covariates did not allow us to examine changes in RAR and dementia risk; (5) internal circadian clocks and environmental factors such as light exposure and social obligations were not assessed or controlled such that it is not possible to separate intrinsic and extrinsic influences on RAR.

### Conclusions

We found that altered daily rest-activity patterns were linked to future risk of dementia or MCI, independent of other known risk factors. Monitoring of ambulatory daily motor activity or rest-activity patterns with wearable devices may provide a unique opportunity to identify people at higher risk of dementia or MCI.

## Appendix

Multimedia Appendix 1 Activity count calculation.

Multimedia Appendix 2 Nonparametric analysis of circadian rest-activity rhythms.

Multimedia Appendix 3 Effects of rest-activity rhythm (RAR) measures on the risk for developing dementia (excluding those with only mild cognitive impairment; MCI). Among those with no dementia or MCI at baseline (N=91,517), 489 participants developed dementia during the follow-up.

Multimedia Appendix 4 Effects of rest-activity rhythm (RAR) measures on risk of developing dementia or mild cognitive impairment (MCI) in non–shift workers.

Multimedia Appendix 5 Effects of rest-activity rhythm (RAR) measures on risk of developing dementia or mild cognitive impairment (MCI) in participants with lower polygenic risk score (PRS) for Alzheimer disease (<median PRS) and participants with higher PRS (>median PRS).

Multimedia Appendix 6 Effects of rest-activity rhythm (RAR) measures on risk of developing dementia or mild cognitive impairment (MCI) in the younger group (<70 years) and older group (≥70 years).

## Abbreviations

HR: hazard ratio
*ICD-10*: *International Classification of Disease, 10th Revision*
IS: interdaily stability
IV: intradaily variability
L5: activity level of the least active 5-hour period
M10: activity level of the most active 10-hour period
MCI: mild cognitive impairment
NHS: National Health Service
PRS: polygenic risk score
Q: quartile
RA: relative amplitude
RAR: rest-activity rhythm
UKB: UK Biobank
