# Supplementary material for: Association of Rest-Activity Rhythm and Risk of Developing Dementia or Mild Cognitive Impairment in the Middle-Aged and Older Population: Prospective Cohort Study
Source: JMIR Public Health Surveill. 2024 May 7;10:e55211. doi: 10.2196/55211 (PMC11109857; doi:10.2196/55211)
Supplement: Multimedia Appendix 1 [file publichealth_v10i1e55211_app1.pdf]

Multimedia Appendix 1. Activity count calculation.

Generation of activity counts.

The activity counts provided by the UK biobank were generated from raw acceleration data using a five-step procedure proposed by Doherty et al.:[1] (1) resampling, (2) calibration, (3) calculating vector magnitude, (4) removing noise and gravity, and (5) generating activity count in each epoch. However, there is a major concern on Step 4 of the procedure, in which one gravitational unit was from the magnitude of each acceleration vector and those negative values were replaced with zeros. This approach could not remove the effect of gravity correctly. Except for the cases when the direction of the acceleration vector is in the same direction of gravity (See Case 1 in **Supplementary Figure 1**), all the derived magnitudes should be smaller than those real values. Such an underestimation will lead to many artificial zeros when the magnitude of derived acceleration vector is smaller than g (the magnitude of gravity) (See Case 2 in **Supplementary Figure 1**), which can occur even when the magnitude of the real acceleration vector is up to 2g (See Case 3 in **Supplementary Figure 1**). The underestimation may impact the assessment of activity patterns during rest or sleep when acceleration is relatively small, and motion is transit (not continuously).

To address the concern, we have modified the step of removing gravity and considered the change in acceleration, $\overrightarrow{\triangle\, a(t_i)}$, (**Supplementary Figure 2**) that automatically removed the gravity. The activity count in an epoch $(t_1, t_2, \ldots, t_n)$ was then calculated as the sum of the magnitudes of all the acceleration change vectors ($|\triangle\, a(t_1)| + |\triangle\, a(t_2)| + \cdots + |\triangle\, a(t_n)|$) in the epoch. In this study, we used the epoch length of 15 seconds.

In addition, we examined the raw acceleration data in those 'stationary periods' (no motion) according to the previously pubication[2]. We found that, though most of the changes in raw acceleration data in each of the three axes were zeros as expected, there were certain values of 4/256 g, 8/256 g, 12/256 g, and even 16/256 g (**Supplementary Figure 3**; note that the resolution is 1/256 g). To minimize the contributions of these noise-related non-zero values to derived activity counts, we included two extra steps: (1) After the first step of resampling and before the step of calibration, we replaced all the acceleration changes of <=4/256 g with zeros (considering that the majorities of noise-related changes in acceleration are 4/256 g). (2) We also examined activity counts in 15-sec epochs generated by our modified procedure. In those 'stationary' and/or 'off-wrist' periods, certain epochs had non-zero activity counts and most of them (over 99%) were <1.5 (**Supplementary Figure 4**). Thus, we used '1.5' as the threshold to filter the activity count recordings, i.e., activity count values < 1.5 were replaced with zeros.

References:

1. Doherty A, Jackson D, Hammerla N, Plötz T, Olivier P, Granat MH, White T, van Hees VT, Trenell MI, Owen CG, Preece SJ, Gillions R, Sheard S, Peakman T, Brage S, Wareham NJ. Large Scale Population Assessment of Physical Activity Using Wrist Worn Accelerometers: The UK Biobank Study. PLoS ONE. 2017;12(2):e0169649. PMCID: PMC5287488

2. van Hees VT, Renström F, Wright A, Gradmark A, Catt M, Chen KY, Löf M, Bluck L, Pomeroy J, Wareham NJ, Ekelund U, Brage S, Franks PW. Estimation of daily energy

expenditure in pregnant and non-pregnant women using a wrist-worn tri-axial accelerometer. PLoS One. 2011;6(7):e22922. PMCID: PMC3146494

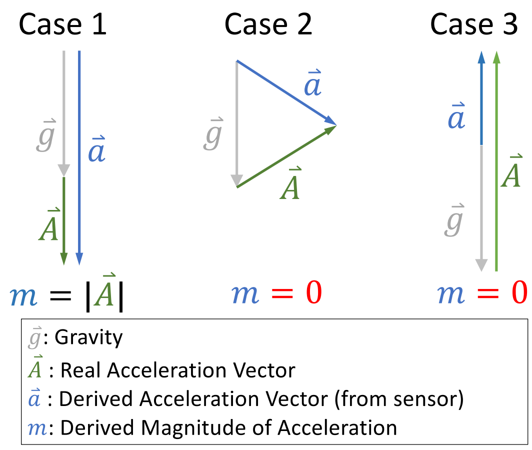

Supplementary Figure 1. Three examples for derived magnitude of acceleration using the algorithm proposed by Doherty et al. (**Case 1**) The real acceleration vector is in the same direction of gravity. (**Case 2**) The real acceleration vector is not in the same direction of gravity, leading to a derived accelerated vector with a magnitude < g. (**Case 3**) The real acceleration vector is in the opposite direction of gravity, leading to a derived accelerated vector with a magnitude < g.

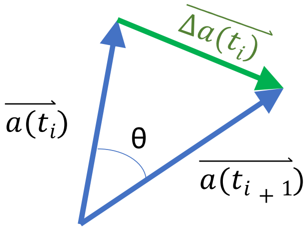

Supplementary Figure 2. Constructed vector for the change in acceleration. Acceleration vectors at two consecutive time point t_i and t_{i+1}, $\overrightarrow{a(t_\iota)}$ and $\overrightarrow{a(t_{\iota+1})}$, are used to obtain the acceleration change vector $\overrightarrow{\triangle\, a(t_\iota)}$.

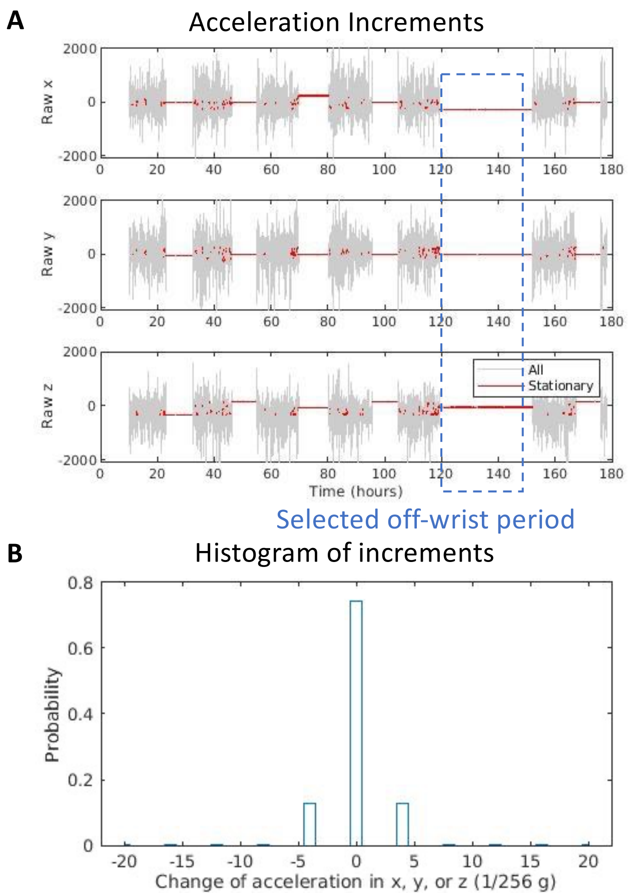

Supplementary Figure 3. An example of raw accelerometer recording. (**A**) Acceleration increments in x, y, and z axes. A long off-wrist period between ~120-150 hours was identified (dashed blue box). (**B**) Histogram of acceleration increments in three directions within the selected long off-wrist period. Probability of dx, dy, or dz > =4/256 was 0.02451%; Probability of dx, dy, or dz >=8/256 was 0.00399%; Probability of dx, dy, or dz >=12/256 was 0.0021%; Probability of dx, dy, or dz > 16/256 was 0.00114%.

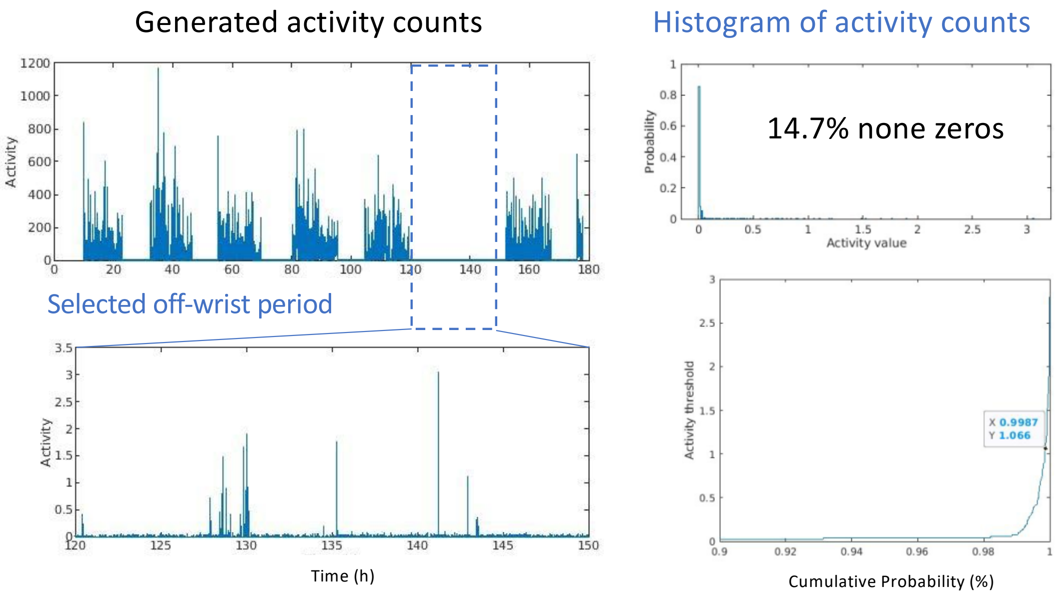

Supplementary Figure 4. Effects of noise on activity counts. (Left Panel) Activity counts generated from raw acceleration data in Supplementary Figure 3A. Within the selected off-wrist period of ~30 hours (dashed box), activity counts in 15-sec epochs (expected to be zeros) are not always zeros due to certain background noise (see Supplementary Figure 3). (Right Panel) Histogram of activity counts (top) and cumulative probability for activity counts up to certain threshold (bottom) in the selected off-wrist period. In those epochs (expected to be zeros), 14.7% had non-zero values, ~99.87% (99.97%) of epochs had activity counts <=1.066 (1.5).
