# Supplementary material for: Association of Rest-Activity Rhythm and Risk of Developing Dementia or Mild Cognitive Impairment in the Middle-Aged and Older Population: Prospective Cohort Study
Source: JMIR Public Health Surveill. 2024 May 7;10:e55211. doi: 10.2196/55211 (PMC11109857; doi:10.2196/55211)
Supplement: Multimedia Appendix 2 [file publichealth_v10i1e55211_app2.pdf]

Multimedia Appendix 2. Nonparametric analysis of circadian rest-activity rhythms.

Mathematical calculations of interdaily stability (IS), intradaily variability (IV) are as below:

$$IS = \frac{n\sum_{h=1}^{24}(\overline{x}_h - \overline{x})^2}{24\sum_{i=1}^{n}(x_i - \overline{x})^2}$$

$$IV = \frac{n\sum_{i=2}^{n}(x_i - x_{i-1})^2}{(n-1)\sum_{i=1}^{n}(x_i - \overline{x})^2}$$

where n is the total number of activity count data points, $\overline{x}$ is the average of the 24-hour activity counts, $\overline{x}_h$ is the hourly average of activity counts, and x_i is the individual activity count data point.
