# Supplementary material for: Association of Rest-Activity Rhythm and Risk of Developing Dementia or Mild Cognitive Impairment in the Middle-Aged and Older Population: Prospective Cohort Study
Source: JMIR Public Health Surveill. 2024 May 7;10:e55211. doi: 10.2196/55211 (PMC11109857; doi:10.2196/55211)
Supplement: Multimedia Appendix 3 [file publichealth_v10i1e55211_app3.pdf]

Multimedia Appendix 3. Effects of rest-activity rhythm (RAR) measures on the risk for developing dementia (excluding those with only mild cognitive impairment [MCI]). Among those with no dementia or MCI at baseline (n=91,517), 489 developed dementia during the follow-up.

RAR characteristics		Fully Adjusted Model
**Relative Amplitude**	Q1	1.80 (1.38-2.35)
	Q2	1.26 (0.95-1.67)
	Q3	0.93 (0.69-1.26)
	Q4	Ref.
	Per 1-SD Decrease	1.23 (1.17-1.30)
**M10**	Q1	1.67 (1.27-2.21)
	Q2	1.10 (0.81-1.48)
	Q3	0.99 (0.72-1.35)
	Q4	Ref.
	Per 1-SD Decrease	1.33 (1.17-1.51)
**M10 midpoint**	Earlier	1.10 (0.89-1.35)
	Middle	Ref.
	Later	1.02 (0.81-1.28)
	Per 1-SD Increase	0.95 (0.85-1.06)
**L5**	Q1	Ref.
	Q2	1.24 (0.96-1.60)
	Q3	0.98 (0.74-1.28)
	Q4	1.44 (1.12-1.85)
	Per 1-SD Increase	1.15 (1.09-1.22)
**L5 midpoint**	Earlier	1.08 (0.87-1.35)
	Middle	Ref.
	Later	0.88 (0.70-1.09)
	Per 1-SD Increase	0.90 (0.83-0.98)
**IV**	Q1	Ref.
	Q2	1.67 (1.27-2.20)
	Q3	1.38 (1.04-1.83)
	Q4	1.51 (1.14-1.99)
	Per 1-SD Increase	1.12 (1.03-1.23)
**IS**	Q1	0.93 (0.72-1.21)
	Q2	0.75 (0.58-0.98)
	Q3	1.04 (0.83-1.31)
	Q4	Ref.
	Per 1-SD Decrease	0.95 (0.86-1.05)
**Amplitude**	Q1	1.88 (1.41-2.50)
	Q2	1.28 (0.94-1.73)
	Q3	1.24 (0.91-1.69)
	Q4	Ref.
	Per 1-SD Decrease	1.38 (1.21-1.58)
**Phase**	Earlier	1.05 (0.85-1.30)
	Middle	Ref.
	Later	0.98 (0.78-1.24)
	Per 1-SD Increase	0.94 (0.85-1.03)

Models are adjusted for age at the time of actigraphy, sex, education, Townsend Deprivation Index, ethnic background, obesity, sleep apnea, morbidity burdens, circulatory disorders, night shiftwork status, alcohol intake, smoking status, and polygenic risk score of for Alzheimer's disease.
