# Supplementary material for: Association of Rest-Activity Rhythm and Risk of Developing Dementia or Mild Cognitive Impairment in the Middle-Aged and Older Population: Prospective Cohort Study
Source: JMIR Public Health Surveill. 2024 May 7;10:e55211. doi: 10.2196/55211 (PMC11109857; doi:10.2196/55211)
Supplement: Multimedia Appendix 5 [file publichealth_v10i1e55211_app5.pdf]

Multimedia Appendix 5. Effects of rest-activity rhythm (RAR) measures on risk of developing dementia/mild cognitive impairment (MCI) in participants with lower polygenic risk score (PRS) for Alzheimer's disease (<median PRS) and participants with higher PRS (>median PRS).

**RAR characteristics**		**Lower PRS**	**Higher PRS**	**P for interaction**
	**Number of participants**	**45,759**	**45,758**	
	**Events of dementia/MCI**	**207**	**348**	
**Relative Amplitude**	Q1	2.06 (1.36-3.12)	1.80 (1.32-2.47)	0.276
	Q2	1.25 (0.80-1.97)	1.32 (0.96-1.83)	
	Q3	1.25 (0.79-1.97)	0.79 (0.54-1.15)	
	Q4	Ref.	Ref.	
	Per 1-SD Decrease	1.27 (1.17-1.38)	1.20 (1.12-1.28)	
**M10**	Q1	2.16 (1.39-3.35)	1.46 (1.05-2.02)	0.164
	Q2	1.03 (0.62-1.68)	1.15 (0.81-1.63)	
	Q3	1.14 (0.69-1.87)	0.98 (0.68-1.41)	
	Q4	Ref.	Ref.	
	Per 1-SD Decrease	1.42 (1.16-1.74)	1.20 (1.08-1.25)	
**M10 midpoint**	Earlier	1.02 (0.74-1.42)	1.15 (0.90-1.47)	0.588
	Middle	Ref.	Ref.	
	Later	1.07 (0.76-1.51)	0.99 (0.75-1.31)	
	Per 1-SD Increase	0.91 (0.77-1.08)	0.93 (0.82-1.06)	
**L5**	Q1	Ref.	Ref.	0.706
	Q2	1.02 (0.69-1.51)	1.38 (1.01-1.88)	
	Q3	0.89 (0.59-1.35)	1.09 (0.79-1.52)	
	Q4	1.38 (0.95-2.01)	1.63 (1.20-2.21)	
	Per 1-SD Increase	1.16 (1.06-1.28)	1.15 (1.08-1.22)	
**L5 midpoint**	Earlier	1.35 (0.95-1.90)	0.93 (0.73-1.20)	0.111
	Middle	Ref.	Ref.	
	Later	1.15 (0.81-1.62)	0.74 (0.57-0.96)	
	Per 1-SD Increase	0.94 (0.82-1.07)	0.90 (0.82-0.99)	
**IV**	Q1	Ref.	Ref.	0.542
	Q2	1.58 (1.02-2.44)	1.68 (1.22-2.31)	
	Q3	1.42 (0.91-2.21)	1.33 (0.95-1.85)	
	Q4	1.81 (1.18-2.76)	1.40 (1.01-1.95)	
	Per 1-SD Increase	1.20 (1.05-1.37)	1.10 (0.99-1.23)	
**IS**	Q1	0.66 (0.43-1.01)	1.25 (0.92-1.69)	0.246
	Q2	0.75 (0.51-1.11)	0.86 (0.63-1.19)	
	Q3	0.86 (0.60-1.22)	1.12 (0.84-1.49)	
	Q4	Ref.	Ref.	
	Per 1-SD Decrease	0.87 (0.75-1.01)	1.05 (0.94-1.17)	
**Amplitude**	Q1	2.25 (1.43-3.52)	1.68 (1.20-2.33)	0.786
	Q2	1.41 (0.87-2.28)	1.23 (0.87-1.75)	
	Q3	1.37 (0.84-2.23)	1.17 (0.81-1.67)	
	Q4	Ref.	Ref.	
	Per 1-SD Decrease	1.45 (1.18-1.78)	1.25 (1.08-1.45)	
**Acrophase**	Earlier	0.85 (0.62-1.18)	1.08 (0.84-1.38)	0.557
	Middle	Ref.	Ref.	
	Later	0.84 (0.59-1.20)	0.94 (0.71-1.25)	
	Per 1-SD Increase	0.94 (0.81-1.09)	0.92 (0.82-1.04)	

Models are adjusted for age at the time of actigraphy, sex, education, Townsend Deprivation Index, ethnic background, obesity, sleep apnea, morbidity burdens, circulatory disorders, night shiftwork status, alcohol intake, and smoking status.
