# Supplementary material for: Association of Rest-Activity Rhythm and Risk of Developing Dementia or Mild Cognitive Impairment in the Middle-Aged and Older Population: Prospective Cohort Study
Source: JMIR Public Health Surveill. 2024 May 7;10:e55211. doi: 10.2196/55211 (PMC11109857; doi:10.2196/55211)
Supplement: Multimedia Appendix 6 [file publichealth_v10i1e55211_app6.pdf]

Multimedia Appendix 6. Effects of rest-activity rhythm (RAR) measures on risk of developing dementia/mild cognitive impairment (MCI) in the younger group (<70 years) and older group (≥ 70 years).

RAR characteristics		Younger participants	Older participants	P for interaction
	**Number of participants**	**74,419**	**17,098**	
	**Events of dementia/MCI**	**242**	**313**	
**Relative Amplitude**	Q1	1.62 (1.12-2.34)	2.24 (1.60-3.14)	0.989
	Q2	1.17 (0.79-1.73)	1.63 (1.15-2.31)	
	Q3	0.97 (0.64-1.47)	1.16 (0.80-1.69)	
	Q4	Ref.	Ref.	
	Per 1-SD Decrease	1.13 (1.04-1.24)	1.25 (1.18-1.33)	
**M10**	Q1	2.12 (1.46-3.06)	1.75 (1.26-2.43)	0.014
	Q2	1.19 (0.79-1.80)	1.32 (0.94-1.85)	
	Q3	0.96 (0.62-1.48)	1.15 (0.81-1.63)	
	Q4	Ref.	Ref.	
	Per 1-SD Decrease	1.33 (1.13-1.57)	1.35 (1.17-1.55)	
**M10 midpoint**	Earlier	0.80 (0.59-1.06)	1.24 (0.94-1.63)	0.387
	Middle	Ref.	Ref.	
	Later	0.63 (0.46-0.87)	1.18 (0.89-1.57)	
	Per 1-SD Increase	0.85 (0.74-0.98)	0.95 (0.84-1.07)	
**L5**	Q1	Ref.	Ref.	0.352
	Q2	0.96 (0.67-1.39)	1.36 (0.98-1.89)	
	Q3	0.84 (0.58-1.22)	1.18 (0.84-1.66)	
	Q4	1.07 (0.76-1.52)	1.61 (1.17-2.21)	
	Per 1-SD Increase	1.05 (0.94-1.17)	1.16 (1.10-1.23)	
**L5 midpoint**	Earlier	1.18 (0.86-1.61)	1.10 (0.85-1.43)	0.495
	Middle	Ref.	Ref.	
	Later	0.97 (0.71-1.33)	0.88 (0.67-1.16)	
	Per 1-SD Increase	0.90 (0.80-1.01)	0.92 (0.83-1.02)	
**IV**	Q1	Ref.	Ref.	0.230
	Q2	1.71 (1.19-2.47)	1.95 (1.38-2.76)	
	Q3	1.29 (0.87-1.90)	1.45 (1.00-2.09)	
	Q4	1.29 (0.88-1.90)	1.98 (1.40-2.80)	
	Per 1-SD Increase	1.07 (0.95-1.22)	1.20 (1.07-1.33)	
**IS**	Q1	0.71 (0.49-1.03)	1.01 (0.74-1.39)	0.436
	Q2	0.81 (0.56-1.15)	0.82 (0.59-1.13)	
	Q3	0.91 (0.65-1.29)	0.92 (0.67-1.26)	
	Q4	Ref.	Ref.	
	Per 1-SD Decrease	0.86 (0.75-0.98)	0.99 (0.88-1.12)	
**Amplitude**	Q1	2.06 (1.42-3.01)	1.92 (1.38-2.68)	0.479
	Q2	1.26 (0.83-1.90)	1.31 (0.92-1.86)	
	Q3	1.17 (0.77-1.78)	1.32 (0.93-1.87)	
	Q4	Ref.	Ref.	
	Per 1-SD Decrease	1.26 (1.07-1.49)	1.44 (1.24-1.68)	
**Acrophase**	Earlier	0.92 (0.68-1.23)	1.05 (0.80-1.39)	0.222
	Middle	Ref.	Ref.	
	Later	0.66 (0.48-0.91)	1.04 (0.79-1.37)	
	Per 1-SD Increase	0.82 (0.72-0.94)	0.96 (0.86-1.08)	

Models are adjusted for sex, education, Townsend Deprivation Index, ethnic background, obesity, sleep apnea, morbidity burdens, circulatory disorders, night shiftwork status, alcohol intake, smoking status, and polygenic risk score of for Alzheimer's disease.
